# The complete mitogenome of *Lophops carinata* (Hemiptera: Fulgoroidea: Lophopidae)

**DOI:** 10.1080/23802359.2021.1944377

**Published:** 2021-07-01

**Authors:** Shi-Yan Xu, Xiang-Sheng Chen

**Affiliations:** a College of Life Science, Guizhou Normal University, Guiyang, Guizhou, China; b Institute of Entomology, Guizhou University, Guiyang, Guizhou, China

**Keywords:** Mitochondrial genome, phylogeny, Hemiptera, Fulgoroidea

## Abstract

In the present study, the complete mitochondrial genome of *Lophops carinata* (Hemiptera: Lophopidae) was sequenced for the first time through next-generation sequencing. The complete mitogenome of *L*. *carinata* is 15,553 bp in length, with the typical gene content and arrangement usually observed in Hexapods. The mitogenome consisted of 13 protein-coding genes, 22 tRNA genes, 2 rRNA genes, and 1 D-loop. The overall nucleotide composition of the mitogenome was 44.6% A, 14.0% C, 8.3% G, and 33.2% T, with an A + T bias of 77.8%. Phylogenetic analyses from 12 Fulgoroidea species by maximum likelihood were consistent and well supported the basal position of Delphacidae, a close affinity among the families Ricaniidae, Issidae, and Flatidae, and a close relationship between Achilidae and Fulgoridae. And *L. carinata* belong to a separate lineage, located in the middle of the phylogenetic tree.

*Lophops carinata* belongs to Lophopidae with planthopper Families Lophopidae (Hemiptera: Fulgoroidea) established by Stål (1866), containing about 42 genera 143 species have been so far described all over the world. Here, we sequenced, assembled, and annotated the complete mitogenome of *L. carinata* from the male voucher. The specimen of *L. carinata* was collected from Wangmo (N 25.17° and E 106.09°), Guizhou, China, on 19 August 2017. Sample were preserved in absolute ethanol and stored at −20°Cin the Institute of Entomology, Guizhou University, Guiyang, China before morphological identification and DNA extraction. Total genomic DNA was extracted from a single individual using the Qiagen TIANGEN Genomic DNA Extraction Kit (TIANGE, Beijing, China) according to the manufacturer’s instructions. Quality of the extracted DNA was checked on 1% agarose gel, sheared by 250–300 bp segments, A- and B-tailed and ligated to Illumina paired-end (PE) adapters. About 350 bp ligated fragments were quality selected on agarose gel and amplified to yield the corresponding short-insert libraries. Subsequently, HiSeq X 10 was used to sequence PE reads and the length of each read was 150 bp. Raw data was adapter clipped and qualified using tools fastx clipper and fastq_quality_filter in FASTAX-Toolkit, respectively. The resultant contigs were annotated using softwares of Geneious Prime (Drummond et al. 2011). Finally, tRNA genes were predicted using the online software, MITOS (Bernt et al. 2013).

The complete mitogenome of *L. carinata* is 15,553 bp in length (Genbank: MT990448) and is AT-rich, with a base composition of 44.6% A, 33.2% T, 8.3% G, and 14.0% C. It contains 37 genes encoding for: 13 protein-coding genes, 22 transfer RNAs, 2 ribosomal RNAs. In addition, it also includes at least one sequence known as the A + T-rich region of variable length, which plays an important role in the process of transcription initiation and replication regulation (Inohira et al. 1997; Osigus et al. 2013). The length of tRNAs ranges from 60 to 69 bp. The size of the two rRNAs are 1191 and 738 bp, respectively.

Phylogenetic analysis using concatenated complete mitogenome datasets, 12 species to understand the evolutionary relationships of *L. carinata* with the other Fulgoroidea families (Figure 1). Using the Maximum likelihood method and GTR substitution model, we found that Fulgoroidea is divided into two groups: Delphacidae and another Fulgoroidea (Ricaniidae, Issidae, Flatidae, Achilidae, Fulgoridae and Lophopidae). *L. carinata* belong to a separate lineage. This finding indirectly verifies the accuracy of the mitochondrial genome sequencing of *L. carinata*, while the phylogenetic tree also supports the monophyly of Lophopidae (Figure 1).

**Figure 1.**
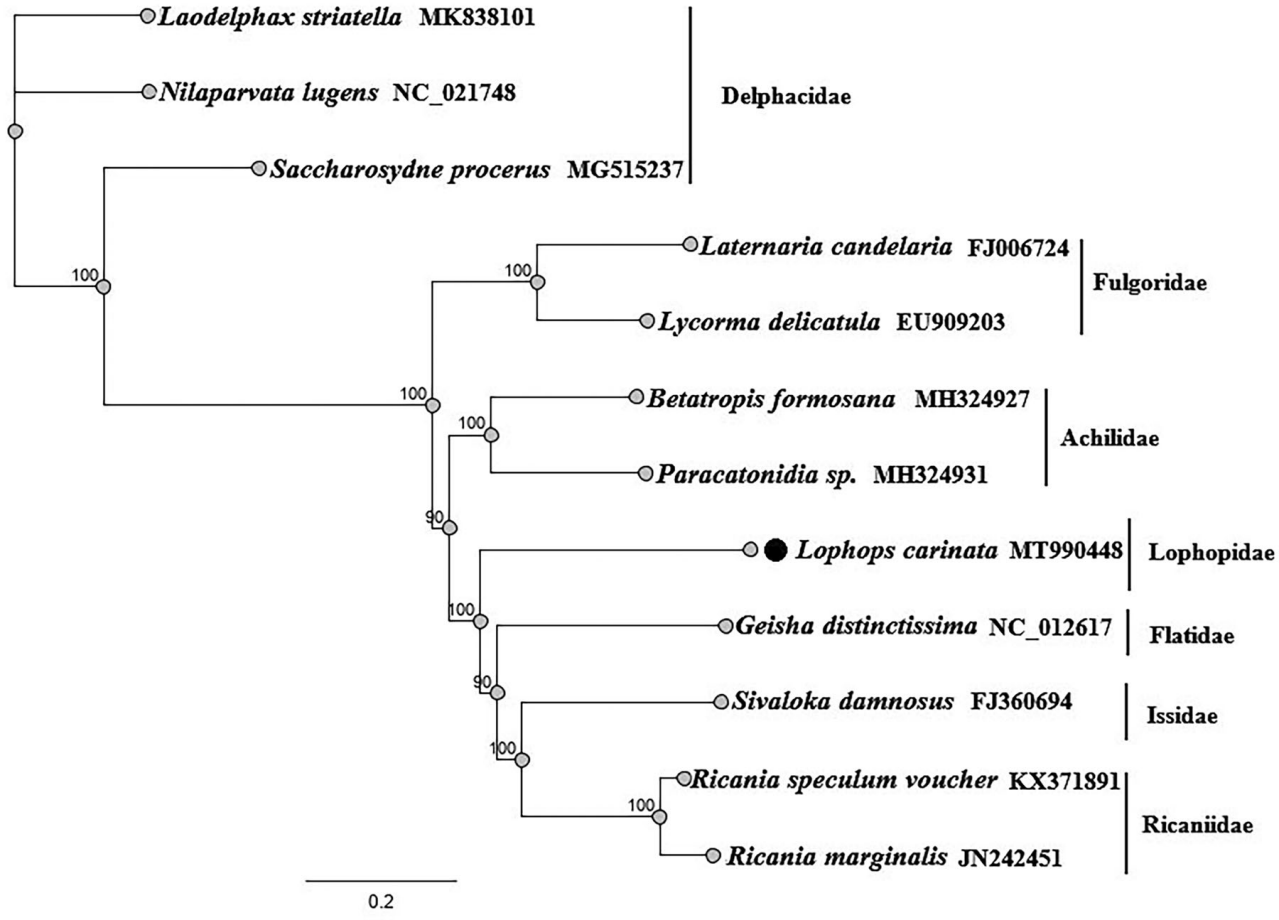
Maximum-likelihood phylogenetic tree inferred from complete mitogenome.

## Data Availability

The data that support the finding of this study are openly available in NCBI at (https://www.ncbi.nlm.nih.gov), reference number [MT990448].
